# Proteomic analysis of swine serum following highly virulent classical swine fever virus infection

**DOI:** 10.1186/1743-422X-8-107

**Published:** 2011-03-08

**Authors:** Jin-fu Sun, Zi-xue Shi, Huan-cheng Guo, Su Li, Chang-chun Tu

**Affiliations:** 1 Institute of Biotechnology, College of Science, Northeastern University, Shenyang 110004, China; 2 Institute of Veterinary Sciences, Academy of Military Medical Sciences, 1068 Qinglong Road, Changchun 130062, China

## Abstract

**Background:**

Classical swine fever virus (CSFV) belongs to the genus *Pestivirus *within the family *Flaviviridae*. Virulent strains of classical swine fever virus (CSFV) cause severe disease in pigs characterized by immunosuppression, thrombocytopenia and disseminated intravascular coagulation, which causes significant economic losses to the pig industry worldwide.

**Methods:**

To reveal proteomic changes in swine serum during the acute stage of lethal CSFV infection, 5 of 10 pigs were inoculated with the virulent CSFV Shimen strain, the remainder serving as uninfected controls. A serum sample was taken at 3 days post-infection from each swine, at a stage when there were no clinical symptoms other than increased rectal temperatures (≥40°C). The samples were treated to remove serum albumin and immunoglobulin (IgG), and then subjected to two-dimension differential gel electrophoresis.

**Results:**

Quantitative intensity analysis revealed 17 protein spots showing at least 1.5-fold quantitative alteration in expression. Ten spots were successfully identified by MALDI-TOF MS or LTQ MS. Expression of 4 proteins was increased and 6 decreased in CSFV-infected pigs. Functions of these proteins included blood coagulation, anti-inflammatory activity and angiogenesis.

**Conclusion:**

These proteins with altered expression may have important implications in the pathogenesis of classical swine fever and provide a clue for identification of biomarkers for classical swine fever early diagnosis.

## Background

Classical swine fever virus (CSFV) is a enveloped, single stranded positive RNA virus of the genus *Pestivirus *within the family *Flaviviridae *[1]. CSFV is the causative agent of classical swine fever (CSF), a highly contagious swine disease and a notifiable disease of the World Organization for Animal Health (OIE).

CSF caused by virulent strains of CSFV is a hemorrhagic disease of pigs, characterized by disseminated intravascular coagulation, thrombocytopenia and immunosuppression. Diseased animals show hemorrhages in the skin, mucosa and internal organs [2,3], and a general immunosuppression featuring a dramatic decrease of peripheral B- and T-cells early after infection of CSFV, due to bystander apoptosis in uninfected cells [4,5].

Studies have shown that cytokines released from monocytes/macrophages activated by CSFV infection may play a critical role in the induction of immune cell apoptosis [6-8], and that proinflammatory and procoagulant cytokines of vascular endothelial cells induced by the virus may disrupt the hemostatic balance and lead to the coagulation and thrombosis seen in acute disease [9]. Proteomic analysis of PK-15 cells *in vitro *and peripheral blood monuclear cells (PBMC) *in vivo *following lethal CSFV infection revealed host cell responses to CSFV infection and changes in protein expression associated with CSFV pathogenesis [10,11].

Apart from above factors that contribute to the pathogenesis and progression of CSF, little is known of changes in serum proteins and biomarker for diagnosis and prognosis of the disease. Recently, growing interest has been focused on the changes in serum protein expression in experimental virus infections to found sera proteins concerning pathogenesis or biomarker for diagnosis or prognosis. Serum contained thousands of protein species secreted and produced from cells and tissues [12,13], which posses rich information concerning overall pathophysiology of the patient or diseased animal [14]. Thereby, analysis of the profile of serum protein alterations is a promising way to try finding potential biomarker and highlighting the pathogenesis of disease.

Here, we report a proteomic analysis of serum protein profile of CSFV-infected pigs and uninfected controls, in which the alterations of protein expression in CSFV-infected pig serum were characterized by two-dimension differential gel electrophoresis (2-D DIGE) followed by MALDI-TOF MS or LTQ MS. A total of 10 differentialy expressed protein spots have been successfully identified. The results shew an altered pattern of protein expression in CSFV-infected pig serum and provide a clue for identification of biomarkers for classical swine fever early diagnosis.

## Results

### Comparative proteomic analysis of CSFV-infected and uninfected serum samples

**Figure 1:**
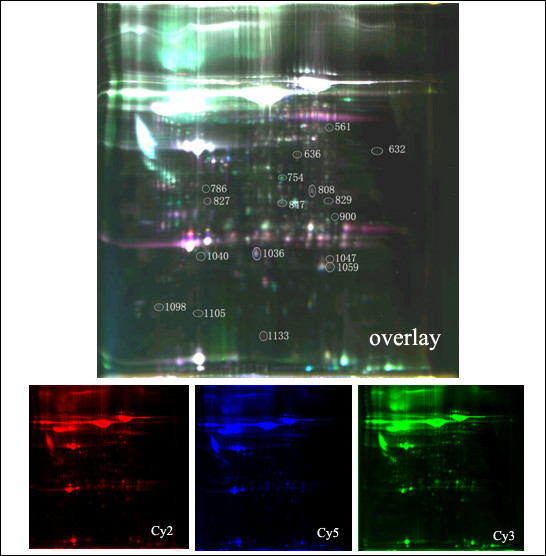
**Representative 2D-DIGE image of serum proteins from control and CSFV-infected pigs**. The image shown is of a 150 μg sample (50 μg each of Cy2-, Cy3- and Cy5-labeled samples) run on a pH 3-10 non-linear gradient IPG strip and 12.5% polyacrylamide gel. Circled and numbered spots in image have been identified as differentially expressed protein spots. Cy2 (red) image of proteins from internal standard samples; Cy3 (green) image of proteins from CSFV-infected sera; Cy5 (blue) image of proteins from control sera.

Serum proteomic profiles of CSFV-infected and uninfected pigs were analyzed by 2-D DIGE. Representative 2-D DIGE profiles of infected, control and internal standard samples are displayed in Figure 1. Images analysis showed that there were between 1127 and 1213 protein spots in each 2D-DIGE gel with 17 spots showing changes of at least 1.5 fold up- or down-regulated expression in infected serum samples (Figure 1). The relative abundance volume ratios of protein spots in CSFV-infected and uninfected serum samples are shown in Table 1, 2.

**Table 1:** Proteins with at least 1.5-fold quantitative alteration in expression in serum from CSFV-infected pigs measured by 2-D DIGE and MALDI-ToF MS analysis

**Spotno**^**.a**^	Identified proteins	**GI no.**^**b**^	**Ratio (I/U)**^**c**^	**MM**^**d **^**(kDa)/pI**	**M/S**^**e**^	**Seq Cov (%)**^**f**^	**Score**^**h**^	*t-*tes *P *value
632	similar to RNA binding motif protein 15B	gi| 126336349	-2.1	99/9.9	7/12	9	71	0.004
636	serotransferrin	gi| 136192	1.5	79/6.9	7/14	15	81	0.003
808	MGF 505-3R	gi| 162849409	1.6	12/9.2	5/16	52	75	0.005
827	vitamin D-binding protein	gi| 51863317	-2.1	25/5.0	9/19	33	126	0.001
1036	retinol-binding protein 4	gi| 47522930	1.7	23/5.4	6/14	31	82	0.004
1098	similar to thrombin inhibitor isoform 2	gi| 119915930	-1.5	42/5.6	5/11	28	78	0.002

a) Labels of protein spots in Figure 1.

b) GI no. is MASCOT results of MALDI-ToF MS from the NCBInr database.

c) Relative protein expression in infected/uninfected samples.

d) MM, Molecular mass.

e) Number of mass values matched/Number of mass values searched.

f) Sequence coverage (%), the percentage of sequence covered *versus *the size of the matched protein.

h) Probability based Mowse score.

**Table 2:** Proteins with at least 1.5-fold quantitative alteration in expression in serum from CSFV-infected pigs by 2-D DIGE and LTQ MS analysis

**Spot no. **^**a**^	Identified proteins	**GI no. **^**b**^	**MM**^**c **^**(kDa)/pI**	**Ratio (I/U)**^**d**^	**Coverage percent(%)**^**e**^	*t-*test *P *value
829	serotransferrin	gi|136192	77/6.9	1.7	4.02	0.009
847	complement c4	gi|38455780	58/6.0	-1.75	9.86	0.004
1040	apolipoprotein A-I	gi|1892	19/7.1	-1.80	11.59	0.002
1105	haptoglobin	gi|47522826	38/6.5	-2.29	3.75	0.004

a) Labels of protein spots in Figure 1.

b) GI no. is SEQUEST results of LTQ MS from the NCBI Suina database.

c) MM, Molecular mass.

d) Relative protein expression in infected/uninfected samples.

e) The percentage of sequence covered *versus *the size of the matched protein.

### Protein identification by MS

**Figure 2:**
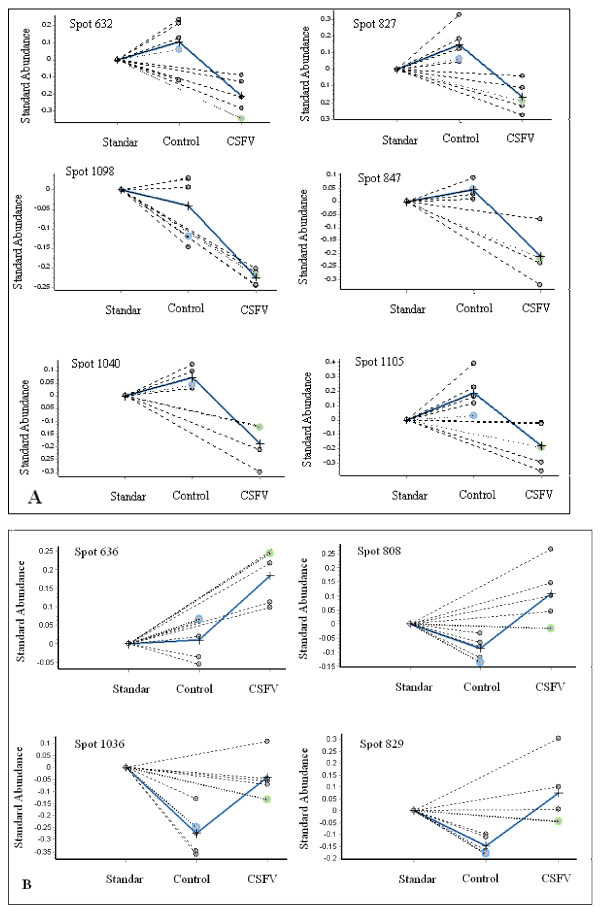
**Graphical representation of protein spots down-regulated (A) and up-regulated (B) in sera from CSFV-infected pigs (p < 0.01)**. Protein spot detection and quantification were performed by the DIA (Differential In-gel Analysis) module of the DeCyder 6.5 software. Each circle represents the abundance of the referred spot in an individual gel, which was expressed as a volume ratio to its corresponding internal standard (spot labeled with Cy2 in the same gel). For each group (control and CSFV-infected pigs), the arithmetic mean of the standardized spot abundances is indicated by the symbol +. To match protein spots across multiple gels and perform statistical analysis (paired Student's *t *test), the BVA (Biological Variation Analysis) module was used.

Of these 17 differentially expressed protein spots, 10 were unambiguously identified by using MALDI-TOF MS or Finnigan LTQ MS (Figure 2, tables 1 and 2), 4 being significantly up-regulated (Figure 2B), and 6 down-regulated (Figure 2A). Morover, two different spots (spot 636 and 829) were identified as one protein of serotransferrin, which showed the presence of isoforms.

## Discussion

In this study, a proteomic method of 2-D DIGE was used to compare serum protein profiles of CSFV-infected pigs and uninfected controls. 2-D DIGE is a fluorescence-based technique for protein visualization and quantification that circumvents the shortcomings of conventional two-dimension polyacrylamide gel electrophoresis (2D-PAGE) such as low sensitivity, reduced dynamic range and gel-to-gel variability [15]. Therefor, 2-D DIGE may present more reliable data with the accurate quantitation and the good reproducibility compared to conventional 2-DE.

A total of 10 protein spots of all 17 differentially expressed protein spots were successfully identified by MS. The remaining 7 protein spots were not identified successfully, either because the quantity was too low to produce a good spectrum or because the confidence levels of the database search using PMF or MS/MS data were insufficient to yield unambiguous results.

Altered expression of proteins in the serum of infected swine is likely a consequence of the infection/disease. How these proteins are implicated in the pathogenesis of CSFV remains unclear, but a brief consideration of their known functions provide some clues.

Both apolipoprotein A-I (apo A-I) and thrombin inhibitor isoform 2 are involved in blood coagulation. Apo A-I participates in the reverse transport of cholesterol, and is believed to have antioxidant and anti-inflammatory effects. Apo A-I is also involved in stimulating endothelial cell movement by high density lipoprotein (HDL) which contributes to the migration of endothelial cells into a wound region and recovery of the endothelium [16]. ApoA-I also takes part in inhibiting the synthesis of platelet-activating factor by endothelial cells [17], and protecting erythrocytes against the generation of procoagulant activity [18]. Thrombin is a plasma serine protease that plays a key role in coagulation and hemostasis but also in thromboembolic diseases. Thrombin inhibitor is a potent inhibitor of thrombin and thrombin-induced platelet aggregation. It is capable of antagonizing host hemostasis and facilitating blood feeding. Down-regulation of apo A-I and thrombin inhibitor isoform 2 may be involved in disruption of hemostatic balance, resulting in the coagulation and thrombosis seen during acute CSF. In addition, down-regulation of apo A-I may affect vascular endothelium repair and inflammatory inhibition.

Another down-regulated protein, haptoglobin (Hp), is a positive acute phase protein. It strongly binds hemoglobin and has anti-inflammatory activities and binds to CD11b/CD18 integrins representing major receptors on the cell membranes of leukocytes [19]. Haptoglobin also has a role in stimulating angiogenesis and has been identified as an angiogenic factor in sera from patients with systemic vasculitis [20]. It is known that CSFV has a particular tropism for vascular endothelial cells in pigs and causes pathological damage. Down-regulation of haptoglobin in CSFV-infected pigs may therefore impede angiogenesis and vascular repair, not facilitate angiogenesis and vascular repair.

Increased retinol-binding protein 4 (RBP4) is a protein bound to some degree to plasma prealbumins, delivering retinol. In plasma, the RBP-retinol complex interacts with transthyretin, which prevents its loss by filtration through the kidney glomeruli. It was reported that RBP4 isoforms and levels were highly increased in the plasma of patients with chronic kidney disease [21], and retinol-binding protein was also identified as a biomarker of acute kidney injury [22]. It is known that CSFV infection may cause kidney petechial bleeding; therefore, upregulation of RBP4 in CSFV-infected pigs may be involved in kidney injury caused by CSFV infection.

Vitamin D-binding protein (VDBP) is a multifunctional protein. In plasma, it carries the vitamin D sterols and scavenges extracellular G-actin released from necrotic cells, inhibiting the formation of polymeric actin filaments (F-actin) that can trigger disseminated intravascular coagulation and multiorgan failure [23]. VDBP also associates with membrane-bound immunoglobulin on the surface of B-lymphocytes and with IgG Fc receptor on the membranes of T-lymphocytes. VDBP is also implicated in macrophage activation and neutrophil chemotaxis [24]. Gasparri et al. have shown that VDBP reduces platelet aggregation and prolongs coagulation time *ex vivo*[25]. Downregulation of multifunctional VDBP in CSFV-infected individuals may therefore be implicated in the pathogenesis of CSFV.

Serotransferrin are iron binding transport proteins, responsible for the transport of iron from sites of absorption and heme degradation to those of storage and utilization. Serotransferrin may also have a further role in stimulating cell proliferation. Drakesmith and Prentice have shown that some virus need iron-replete host for efficient replication and viral infection alters the expression of proteins involved in iron homeostasis, such as HIV-1 and hepatitis C virus infection [26]. Up-regulation of serotransferrin in CSFV-infected serum may implicate that iron-replete homeostasis of host is needed for CSFV efficient replication.

## Conclusion

This is the first report on quantitative serum protein profiles of CSFV-infected pigs. Ten proteins with significant changes in expression have been identified during the acute phase of CSF; further studies focusing on the functional properties and predictive value for CSFV infection may permit identification of biomarkers for early diagnosis of CSFV infection and development of new diagnostic methods.

## Materials and methods

### CSFV infection and serum proteins preparation

Ten 60-day-old Landrace pigs were used. All pigs were free from CSFV infection, as validated by using a protocol described by Shi et al. [27]. Five pigs were injected intramuscularly with a lethal dose of highly virulent CSFV strain Shimen using a previous protocol [27], the remaining 5 serving as uninfected controls.

In the early stage of acute infection, when rectal temperature increased (≥40°C) but without other CSF clinical symptoms, blood samples from each pig were collected and sera were separated. Serum albumin and immunoglobulin (IgG) were removed using a ProteoSeek™ Albumin/IgG Removal Kit (Pierce) according to the manufacturer's instructions. Samples were centrifuged at 12000 g in a YM-3 centrifugal filter (Millipore) to make protein concentration to 5-10 μg/μL as measured using Coomassie Plus-The Better Bradford™ Assay kit (GE Healthcare).

### 2D-DIGE

Treated serum samples were diluted to give stock solutions with final protein concentrations of about 5 mg/mL using DIGE lysing solution (7 M urea, 2 M thiourea, 62 mM Tris, 4%CHAPS, 0.2% IPG buffer, PH8.8). Equal volumes of serum from each infected pig and the control pigs were mixed as an internal standard. Serum samples were labeled with cyanine dyes (GE Healthcare) according to the manufacturer's directions. Briefly, 50 μg of serum samples were minimally labeled with 400 pmol CyDye following the cross-label rule (Cy2 labeling the internal standard, Cy3 and Cy5 cross labeling infected or control samples). Differentially labeled samples (50 μg each of Cy2-, Cy3- and Cy5-labeled samples) were mixed and diluted with rehydration buffer (8 M urea, 4% CHAPS, 130 mM DTT, 2% Pharmalyte, pH 3-10 non-linear) to 350 μl, and loaded onto IPG strips (18 cm; pH 3-10 NL) with active rehydration (30 V for 10 h). Protein components were resolved by first-dimension isoelectric focusing (IEF) conducted at 20°C in an Ettan IPGphor Isoelectric Focusing System I (GE Healthcare), with the current limited to 50 mA/strip and the following voltage program: 200 V/1 h, 500 V/1 h, 1000 V/1 h, linear ramp to 8000 V over 1 h, then 8000 V constant for a total focusing time of 80 000 Vh. The IPG strips were then equilibrated by soaking first for 15 min in 50 mM Tris-HCl (pH 8.8), 6 M urea, 2% SDS, 30% glycerol, 2% (w/v) DTT and a trace of bromophenol blue, then for 15 min in the same solution but containing 2.5% (w/v) iodoacetamide instead of DTT. All strips were subsequently loaded on 12.5% homogeneous SDS polyacrylamide gels and the second-dimensional separation was performed at 20°C at a constant current of 15 mA/gel for 30 min, then 30 mA/gel until the dye reached the bottom of the gel in the Ettan DALT Six System (GE Healthcare).

Unlabeled serum samples (800 μg) were separated by conventional 2-DE, following which the 2-D gels were stained with CBB-G250 (Sigma).

### Image acquisition and analysis

A total of 5 DIGE gels were scanned using a Typhoon 9410 imager (GE Healthcare). Image analysis and statistical analysis were performed with DeCyder 6.5 (GE Healthcare). An internal standard, consisting of an equivolume pool of all samples in the experiment, was run in each gel. Measurement of the relative change of each sample to its internal standard effectively removed gel-to-gel variation, resulting in accurate quantitation of changes between samples (DeCyder 6.5 manual, Section 1.4). Proteins presenting as spots with ≥1.5-fold expression change between the sera from CSFV-infected and control swine were identified as being significantly altered (p ≤ 0.01, Student's *t*-test).

### Protein identification by MS

Changes observed in 2D-DIGE images were matched with serum protein patterns in CBB-stained gels. Spots of interest were excised manually and subject to destaining and trypsin digestion according to the protocol described by Wan et al. [28]. After destaining and trypsin digestion of each protein spot, the peptide mixtures were extracted with extraction solution, 50% acetonitrile/0.5% trifluoroacetic acid (TFA), at 37°C for 1 h and lyophilized for further identification by MS. Peptide extracts were analyzed by MALDI-TOF MS or Finnigan LTQ MS (ThermoQuest) coupled with a Surveyor HPLC system (ThermoQuest). For MALDI-TOF MS analysis, the peptide powders were re-dissolved in 5 μl 0.1% TFA. One μl of supernatant was spotted on anchorChip targets (Bruker Daltonics), and then mixed with 0.5 μl matrix solution (10 mg/ml α-cyano-4-hydroxycinnamic acid in 50% acetonitrile/0.1% TFA). After the sample spot drying, the acquisition of the MALDI spectra was performed on a Bruker autoflex (Bruker Daltonics) MALDI-TOF mass spectrometer operated in reflector mode. The spectra were calibrated using a recently described procedure relying on external calibration followed by internal mass correction. Protein identification was performed by searching the NCBInr database [http://www.ncbi.nlm.nih.gov/ 20080410 (6417748 sequences; 2190362656 residues)] using the MASCOT database search engine. Up to one missed cleavage is permitted and 150 ppm peptide mass error tolerance was used for all tryptic-mass searches. Fixed modifications of carbamidomethylation and variable modifications of oxidation were allowed. For LTQ MS, a microcore RP column (C18 150 μm × 120 mm; Thermo Hypersil, San Jose, CA, USA) was used to separate the peptide extracts. Mobile phase A (0.1% formic acid in water) and mobile phase B (0.1% formic acid in ACN) were selected. The tryptic peptide mixtures were eluted using 2% solvent B for 15 min, increasing linearly to 98% solvent B for 90 min. The peptides were eluted from the C18 microcapillary column at a flow rate of 150 μl per min and then electrosprayed directly into a mass spectrometer (spray voltage 3.2 KV) with a capillary temperature of 170°C. The full scan range was from M/Z 400 to 2000. The data sets obtained in the LC-MS/MS analyses were used for database searches with the SEQUEST search engine (Thermo Electron) against the database of NCBI Suina. A relative molecular mass of 57 Da was added to the average molecular mass of cysteines in MS/MS data searching. Both *b *ions and *y *ions were included in the database search. Protein identification results were filtered with Xcorr(1 + ≥ 1.9, 2 + ≥ 2.2, 3 + ≥ 3.75) and DelCn(≥ 0.1).

## Abbreviations

CBB: Coomassie brilliant blue; CHAPS: 3-[(3-cholamidopropyl) dimethyl-ammonio]-1-propanesulfonate; DIGE: difference gel electrophoresis; DTT: dithiothreitol; IPG: immobilized pH gradient; MALDI-TOF MS: matrix-assisted laser desorption/ionization time-of-flight mass spectrometry; 2-DE: two-dimensional electrophoresis

## Competing interests

The authors declare that they have no competing interests.

## Authors' contributions

JS performed the experiments, analyzed data and wrote manuscript; ZS and HG inoculated pigs with CSFV, prepared serum samples and performed 2D DIGE; SL conducted image analysis. CT is the supervisor of the study group, designed the experiments and wrote the manuscript. All authors read and approved the final manuscript.
